# Sources of water vapor and their effects on water isotopes in precipitation in the Indian monsoon region: a model-based assessment

**DOI:** 10.1038/s41598-023-27905-9

**Published:** 2023-01-13

**Authors:** Thejna Tharammal, Govindasamy Bala, Jesse M. Nusbaumer

**Affiliations:** 1 Centre for Atmospheric and Oceanic Sciences, Indian Institute of Science, Bangalore, India; 2 National Center for Atmospheric Research, Boulder, CO USA; 3 Present Address: Interdisciplinary Centre for Water Research, Indian Institute of Science, Bangalore, India

**Keywords:** Climate sciences, Hydrology

## Abstract

Climate records of ratios of stable water isotopes of oxygen (δ^18^O) are used to reconstruct the past Indian monsoon precipitation. Identifying the sources of water vapor is important in understanding the role of monsoonal circulation in the δ^18^O values, to aid in monsoon reconstructions. Here, using an isotope-enabled Earth system model, we estimate the contributions of oceanic and terrestrial water vapor sources to two major precipitation seasons in India—the Southwest monsoon and the Northeast monsoon, and their effects on the δ^18^O in precipitation (δ^18^O_p_). We find that the two monsoon seasons have different dominant sources of water vapor because of the reversal in atmospheric circulation. While Indian Ocean regions, Arabian Sea, and recycling are the major sources of the Southwest monsoon precipitation, North Pacific Ocean and recycling are two crucial sources of Northeast monsoon precipitation. The δ^18^O_p_ of the Southwest monsoon precipitation is determined by contributions from the Indian Ocean sources and recycling. Despite reduced precipitation, more negative δ^18^O_p_ values are simulated in the Northeast monsoon season due to larger negative δ^18^O_p_ contributions from the North Pacific. Our results imply that changes in atmospheric circulation and water vapor sources in past climates can influence climate reconstructions using δ^18^O.

## Introduction

Stable isotopes of water are used as proxies to reconstruct the past Indian monsoon precipitation based on the climate-dependent fractionation of the water molecule. Monsoon reconstructions using isotope proxy archives rely on the “amount effect”^1^, the empirical inverse relationship between the amount of precipitation at a location, and the ratios of oxygen isotopes (ratio of heavier to lighter isotope, R, ^18^O/^16^O) in precipitation, frequently referred to as “water isotopes”. The water isotopic composition is denoted by the delta (δ) value in units of permil (‰) and for a sample is commonly calculated relative to the Vienna standard mean ocean water (VSMOW).

$${\mathrm{For \; oxygen},\delta }^{18}O=\frac{{R}_{sample}}{{R}_{VSMOW}}-1\times 1000.$$

The amount effect is generally explained by the continued condensation and rainouts from an air mass during heavy precipitation events that deplete the vapor of heavier isotopes^1,2^. The advancement of isotope-enabled climate and Earth system models^3^ has improved our understanding of the effects of various climate factors on the water isotope ratios in precipitation [δ^18^O_p_]^3^. Studies have shown that the δ^18^O_p_ values in a region are also influenced by changes in circulation^4^, upstream rainout processes, and water vapor source location^5–8^. For instance, interpretations of δ^18^O records from the Chinese speleothems as an indicator for East Asian summer monsoon precipitation are debated, as, these records may be controlled by water vapor sources or transportation pathways, than the local precipitation amount^5,6,9,10^. This highlights the importance of understanding the effects of circulation changes and water vapor sources on the δ^18^O_p_ values in the Indian monsoon region. A few observational studies have attempted to distinguish the effects of water vapor sources on the water isotopes in the Indian monsoon precipitation^11–14^. However, comprehensive studies on the effect of water vapor sources on the δ^18^O_p_ in the Indian monsoon region using isotope-enabled Earth system models have been limited.

The Indian summer monsoon system (southwest-SW monsoon; during June–July–August–September, JJAS) is a coupled ocean–atmosphere phenomenon, caused by the migration of the intertropical convergence zone (ITCZ) to the northern hemisphere in the summer, and is associated with intense low-level southwesterly winds^15^. Summer monsoon precipitation amounts to ~ 80% of the annual precipitation in India and supports agriculture and livelihood in one of the most populous regions in the world. Besides the SW monsoon season, the monsoon system comprises the northeast winter monsoon (NE) season (from October to December, OND), when the lower-level circulation over India reverses direction from southwest to northeast because of the southward movement of ITCZ^16,17^. Although the NE monsoon contributes only ~ 11% of the annual precipitation in India, it is important in the southern peninsula where it provides ~ 30–60% of the annual precipitation^16^. Changes in atmospheric circulation between the SW and NE monsoon seasons can provide a climate analog to test the effect of changes in circulation and water vapor sources on the δ^18^O_p_ values in the Indian region.

Further, climate-modeling studies on the sources of water vapor for the Indian dual monsoon precipitation are lacking. A few earlier global studies^18–20^ using water vapor tagging in climate models have identified the importance of Indian Ocean sources and precipitation recycling for South Asian precipitation. Most of the studies that identify water vapor sources of monsoon precipitation rely on analytical models and Lagrangian techniques^21–25^. Such trajectory estimates, however, have drawbacks due to simplified calculations and sensitivity to uncertainties in the reanalysis data^21,26^. Identification of water vapor sources for continental precipitation is crucial in climate change studies. The inclusion of water vapor tracking in climate models would bring clarity on source effects on isotope ratios and past monsoon reconstructions, and studies in this direction using Earth system models are important for the Indian region. Previous studies^21–25^, using the Lagrangian trajectory approach, and a climate modeling study^27^ identify the Indian Ocean, the Arabian Sea, and precipitation recycling^23,28^ as the major sources of the SW monsoon precipitation. Studies on water vapor sources of NE monsoon precipitation are rare, although trajectory analysis^22^ and observational isotope tracking^13^ suggest precipitation recycling and Bay of Bengal are important sources in this season.

The recently developed water isotope-enabled Community Earth System Model, iCESM1^29^, can track the sources of water vapor and isotopes by tagging their evaporative fluxes, besides simulating water isotopic ratios in the model hydrology. Here, we focus on identifying the major sources of water vapor for the Indian summer and winter monsoon precipitation and the effects of the reversal of monsoon circulation on the δ^18^O_p_ values, which will have implications for paleo-monsoon reconstructions. Our results are based on climate simulations using the iCESM1 forced by prescribed sea surface temperatures (Supplementary Fig. S1) and sea ice concentrations from 1979 to 2003. The evaporative fluxes of the oxygen isotope species and water vapor from 16 oceanic and terrestrial regions around the Indian subcontinent are tracked. See the “Methods” section for details on the model simulation and methodology.

## Results

### Seasonal monsoon circulation and precipitation

**Figure 1:**
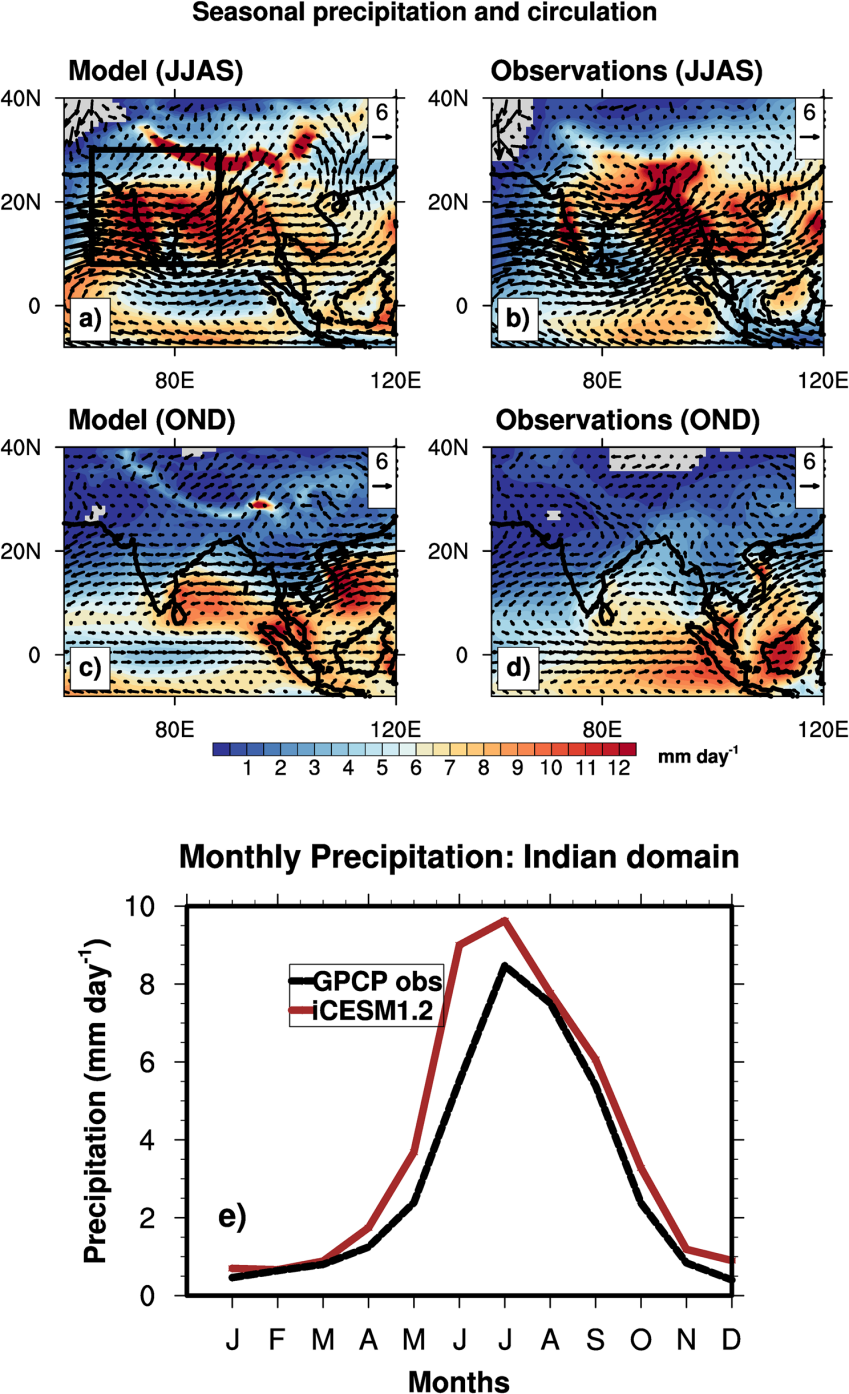
Modeled and observational precipitation in mm/day and 850 hPa winds over India in the JJAS and OND seasons. Precipitation observations are from GPCP, and winds are from ERA5 reanalysis over the years 1980–2000. Model data is averaged from 1980 to 2003, and GPCP data is the long-term (1980–2010) mean. Precipitation rates over land that are less than 0.1 mm/day are not shown. Panels (**a**,**c**) Modeled JJAS and OND seasons’ precipitation rate and 850 hPa winds. Panels (**b**,**d**) Corresponding long-term mean precipitation rates and 850 hPa winds from the GPCP and ERA5 data, respectively. Panel (**e**) Monthly mean simulated and GPCP precipitation data averaged over the land grid cells in the Indian domain (8° N–30° N, 65° E–88° E; Black box in panel **a**). The figures were created using NCAR Command Language (NCL) Version 6.6.2 (http://www.ncl.ucar.edu/).

Figure 1 shows the spatial distribution of simulated climatological (mean of years 1980–2003) SW and NE monsoon circulations and precipitation, and the monthly mean precipitation over the Indian monsoon domain (8° N–30° N, 65° E–88° E). The model successfully reproduces the reversal of circulation between the monsoon seasons (Fig. 1a,c) and seasonal precipitation in the Indian domain, when compared to the observations (Fig. 1b,d; GPCP^31^ precipitation climatology and winds from ERA5 reanalysis^32^). The SW monsoon circulation is distinguished by the strong westerly winds at 850 hPa from the Indian Ocean to the Indian land mainly due to differential heating of land and ocean (Supplementary Fig. S2). Maximum precipitation in the Indian region is simulated in the SW monsoon season (~ 8 mm/day domain mean in India) with larger precipitation rates in the tropical latitudes, except in the southeastern peninsular region. The model slightly overestimates the summer precipitation when compared to the observations. Previous studies^33–36^ have also identified these biases in SW monsoon precipitation by CESM, and have attributed them to factors such as convective parameterizations, model resolution, biases in simulated SST, and circulation. The NE monsoon season is distinguished by the reversal of the lower-level circulation from southwesterly to northeasterly. The NE monsoon precipitation is largely confined to southeastern India, and the simulated domain mean over India is ~ 2 mm/day, which is slightly greater than the observations (Fig. 1e). In general, simulated seasonal precipitation rates are in agreement with the observations and previous model simulations^34^.

### Major water vapor sources of the seasonal precipitation

**Figure 2:**
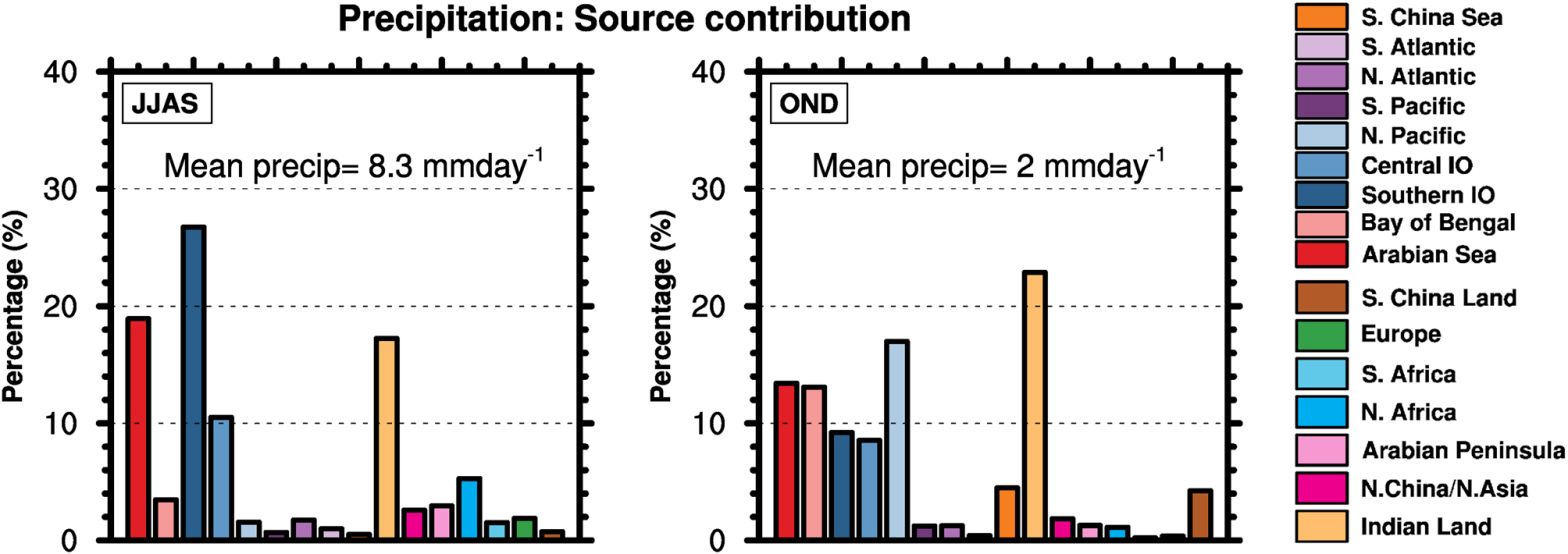
Relative contributions of the regional sources in percentage to the mean seasonal precipitation (domain mean of P_tag/_P_total_) in the Indian domain. Left) regional source contributions to the SW monsoon precipitation, Right) same as left, but for the NE monsoon precipitation. Indian domain means of precipitation rates for both seasons are shown inside the panels. The figures were created using NCAR Command Language (NCL) Version 6.6.2 (http://www.ncl.ucar.edu/).

Figure 2 shows the relative contributions (domain mean of P_tag_/P_total_ over India, P_tag_ is the contribution to precipitation from each of the tagged regions, and P_total_ is net precipitation) of the tagged regions to seasonal precipitation. The sum of precipitation contributions from the tags matches the simulated seasonal precipitation (Supplementary Figs. S3 and S4). Dominant sources of precipitation during the SW monsoon season are the Southern and central Indian Ocean regions (38% together), and the Arabian sea (19%), because of the strong low-level southwesterly circulation that transports water vapor from these sources to the land (Fig. 1). Together with precipitation recycling (17%), the above sources contribute ~ 75% of the total precipitation in the summer monsoon season, in agreement with water vapor tracking studies^21,22,24,25^ that identify the Indian Ocean and the Arabian sea as major sources of the SW monsoon precipitation. The considerable contribution of precipitation recycling to the SW monsoon precipitation is consistent with previous water vapor tracking studies^23,25,27,28^, although the modeled estimates are somewhat lower than these previous estimates (~ 20–40%). Increased surface temperature (Supplementary Fig. S2), and abundant monsoon precipitation that enhances the soil water content in the summer months are favorable for recycling. Similar to the precipitation contribution, the Southern Indian Ocean contributes maximum to the lower-level water vapor in the Indian domain (Supplementary Fig. S5), followed by the Arabian Sea and precipitation recycling.

The reversal of the atmospheric circulation in the NE monsoon season leads to changes in the water vapor source location and relative source contributions to the precipitation over India (Fig. 2). The dominant contributor of water vapor to the NE Monsoon precipitation is recycling (~ 23%), due to reduced contributions from the Indian Ocean sources as the circulation is mainly from the north to the south because of colder land masses in the north (Supplementary Fig. S2; Fig. 1). However, the net precipitation from recycling is only ~ 1/4th the value of its SW monsoon contribution, due to smaller precipitation (~ 2 mm day^−1^) in the NE monsoon season. The North Pacific Ocean contributes 17%, due to the intensified easterly circulation from the western Pacific, which is relatively warmer than the land region during this season (Supplementary Fig. S2). In addition to these two sources, the Arabian Sea and the Bay of Bengal (~ 13% each) and Southern and Central Indian Ocean sources (9% each) contribute ~ 84% of the total precipitation in the NE monsoon season. Studies examining the source contributions of NE monsoon precipitation are few compared to the SW monsoon season for a meaningful comparison. In agreement with the results, a trajectory analysis^22^ suggests precipitation recycling as the principal source of October precipitation, while observational studies^13,22,37^ identify the Bay of Bengal as an important source of NE monsoon precipitation. The NE monsoon season has considerably reduced water vapor content and a shorter vertical extent of the water vapor profile in the Indian domain compared to the SW monsoon (Supplementary Fig. S5), due to lower rates of convection and uplifting of vapor in the NE monsoon season^38^.

### Simulated seasonal distributions of δ^18^O_p_

**Figure 3:**
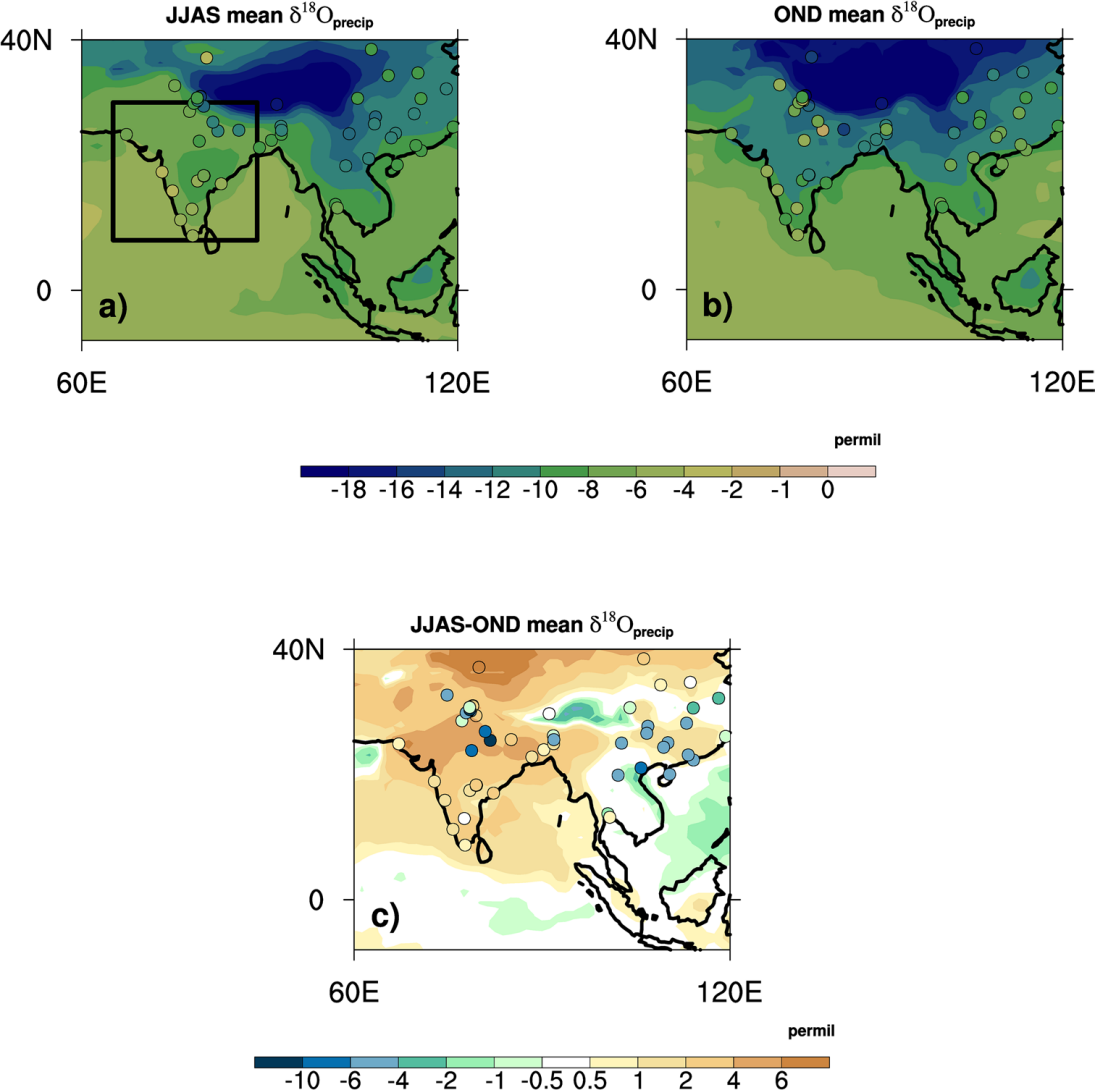
Modeled (color shading) and observational (long-term monthly mean station data from the Global Network of Isotopes In Precipitation-GNIP-filled circles overlaid on the map) δ^18^O_p_ in units of ‰ in the (**a**) JJAS and (**b**) OND seasons (Indian domain is shown in the black box in panel **a**). The simulated differences in precipitation-weighted seasonal δ^18^O_p_ values [between the JJAS and OND seasons] over India in ‰ are shown in panel (**c**). The filled circles in panel (**c**) are JJAS minus OND values calculated from the GNIP data. The figures were created using NCAR Command Language (NCL) Version 6.6.2 (http://www.ncl.ucar.edu/).

The model-simulated precipitation-weighted seasonal δ^18^O_p_ values in the SW and NE monsoon seasons are shown in Fig. 3 and are compared with precipitation-weighted station data from the ‘Global Network of Isotopes in Precipitation (GNIP)’^39^. The model simulates the seasonal distributions of δ^18^O_p_ values in India reasonably well when compared to the observations, especially in the tropical Indian region (8° N–20° N). Previous studies^7^ have also shown that iCESM results are in good agreement with proxy-based South Asian monsoon isotopic variability at longer timescales. The simulated δ^18^O_p_ values are generally more negative than the observations in the Indian monsoon domain in both seasons. However, the number of GNIP stations in the study region is limited, and long-term records for the seasons are lacking for many of the stations. Previous studies using iCESM^40^, and an earlier version of the same isotope model^41^ simulate a similar depletion bias in the tropical monsoon region and suggest it is mainly due to the wet bias (Fig. 1) in the model. Both the model and observations show that the δ^18^O_p_ values of SW monsoon precipitation in the Indian monsoon domain (especially in the tropical latitudes) are more positive than the NE monsoon values (Fig. 3c, domain mean difference of + 2.9‰ between the SW [− 7.6‰] and NE [− 10.5‰] monsoon seasons). This is unexpected, given that the amount of precipitation in the SW monsoon season in the domain is approximately four times greater than the NE monsoon precipitation. These results for the seasonal difference in the δ^18^O_p_ values agree with station-based observations of δ^18^O_p_ from Southern India^12,13^, which suggest the influence of water vapor sources on this difference.

### Source effects on the water isotope ratios of seasonal precipitation

**Figure 4:**
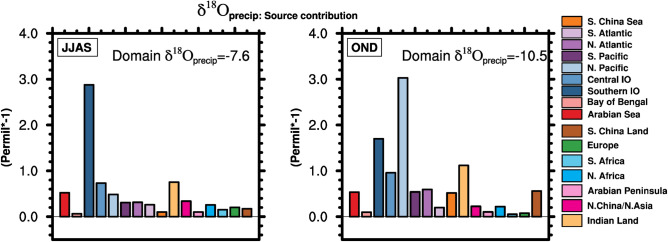
δ^18^O_p_ of water tags (units of permil [‰] and the values are uniformly multiplied by − 1) from different source regions that contribute to the (left panel) Southwest monsoon season precipitation in the Indian domain and (right panel) to the Northeast monsoon season. Domain means of net δ^18^O_p_ (units of ‰) for both seasons are shown inside the panels. The figures were created using NCAR Command Language (NCL) Version 6.6.2 (http://www.ncl.ucar.edu/).

**Figure 5:**
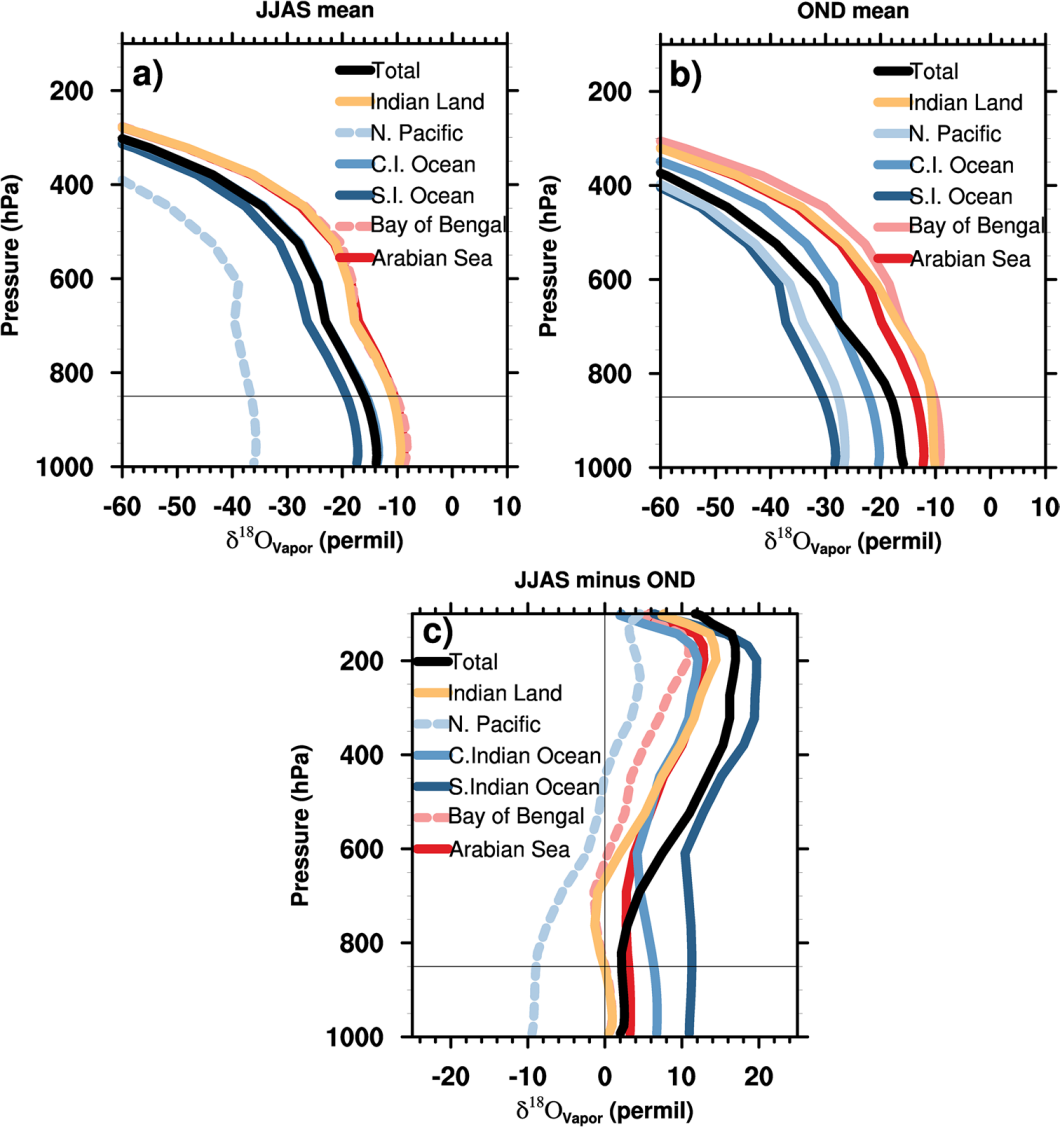
Vertical profiles (in hybrid sigma pressure levels) of the δ^18^O values in water vapor (averaged over the Indian domain) for (**a**) JJAS (**b**) OND and (**c**) JJAS minus OND in units of permil, ‰, from six major sources in the seasons. Dashed lines in panels a and c are non-dominant water vapor contributors (< 10% to precipitation) in the JJAS season (the Bay of Bengal and the North Pacific). Horizontal lines in the panels denote the 850 hPa level. The figures were created using NCAR Command Language (NCL) Version 6.6.2 (http://www.ncl.ucar.edu/).

The net δ^18^O_p_ value in the Indian region can also be calculated as the sum of the individual δ^18^O_p_ contribution from the 16 tags, weighted by their contribution to the total precipitation, as

$${\delta }^{18}{O}_{p}={\sum }_{tag=1}^{tag=16}{\delta }^{18}{O}_{ptag}\times \frac{{P}_{tag}}{{P}_{total}},$$

where δ^18^O_ptag_ is the contribution of δ^18^O_p_ values from each of the tagged regions at the sink region. The precipitation-weighted sum of the δ^18^O_ptag_ values from 16 tags comprises ~ 95% of the simulated δ^18^O_p_ in the Indian region (Supplementary Figs. S3, S4). Figures 4 and 5 respectively show the contributions of the source regions to the δ^18^O_p,_ and the vertical profiles of δ^18^O values in water vapor of major sources (δ^18^O_vtag_) during both seasons in the Indian domain. The Southern Indian Ocean (− 2.8‰), Central Indian Ocean (− 0.7‰), precipitation recycling (− 0.7‰), and the Arabian Sea (− 0.5‰) are the major contributors to the δ^18^O_p_ values in the Indian domain in the summer monsoon season. The δ^18^O_vtag_ values of both Indian Ocean sources are highly depleted compared to those of the Arabian sea and precipitation recycling (~ − 9‰ difference in the lower troposphere).

In contrast to the SW monsoon season, the North Pacific Ocean is the dominant contributor (− 3‰) to the δ^18^O_p_ in the NE monsoon season. Although precipitation recycling is the major contributor to the NW monsoon precipitation, the δ^18^O_ptag_ of the recycling (− 1.1‰) does not differ greatly from the SW monsoon season. Other major contributors to the net δ^18^O_p_ in the NE monsoon are the Southern Indian Ocean (− 1.7‰, + 1.1‰ compared to JJAS value), the Central Indian Ocean (− 0.9‰, depletion by 0.2‰ from the JJAS value), and the Arabian Sea (− 0.5‰, similar to the JJAS value), while the δ^18^O_ptag_ value of the Bay of Bengal is relatively positive (~ − 0.1‰). The δ^18^O values in water vapor of the North Pacific source are more negative than most of the other major sources, whereas, the lower-tropospheric δ^18^O_vtag_ values of the recycling are closely similar to those in the SW monsoon season. The δ^18^O_vtag_ values of the Southern Indian Ocean and the Arabian Sea are comparatively more negative in the NE monsoon season. However, the δ^18^O_ptag_ contributions from these sources are either more positive in the NE monsoon (for the Southern Indian Ocean) or similar to that in the SW monsoon season (for the Arabian Sea), indicating the effect of reduced precipitation from the sources or enrichment of the condensate likely due to post-condensation processes. To further understand the characteristics of δ^18^O_ptag_ of individual major sources in the seasons, we decompose^7^ each of the δ^18^O_ptag_ values of major sources to the effects of source composition, rainouts, and condensation in the following section.

### The effects of condensation, rainouts, and source composition on the δ^18^O_ptag_ of major sources

δ^18^O_ptag_ values at the sink (Indian domain) can be decomposed as the precipitation-contribution weighted sum of effects of (i) condensation at the sink, (ii) rainouts on the trajectory, and (iii) source composition of the vapor^7^. We calculate these three terms for the major sources (> ~ 10% contribution to precipitation in the corresponding season) in the SW and NE monsoon seasons (see “Methods”) and are shown in Table 1.

**Table 1:** Climatological means of δ^18^O_ptag_ [in ‰] from the major source regions at the Indian domain as the precipitation weighted sum of three processes (i) condensation, (ii) rainout, and (iii) source composition (see “Methods”). Major water vapor sources (near to 10% or more contribution in the corresponding season) are shown in the table with the columns in decreasing order of contribution [e.g. SIO in JJAS is the largest contributor]. *SIO* Southern Indian Ocean, *AS* Arabian Sea, *LND* Precipitation recycling, *CIO* Central Indian Ocean, *NPAC* North Pacific, *BOB* Bay of Bengal.

	SW summer monsoon (JJAS)				NE winter monsoon (OND)					
	SIO	AS	LND	CIO	LND	NPAC	AS	BOB	SIO	CIO
Domain mean of P_tag_/P_total_ (Fraction)	0.27	0.19	0.17	0.10	0.23	0.17	0.13	0.13	0.09	0.09
(i) Condensation term (‰)	5.68	5.88	5.08	5.79	5.18	4.63	6.86	7.43	5.15	7.31
(ii) Rainout term (‰)	− 3.83	− 0.19	− 0.07	− 3.22	− 0.20	− 11.59	− 2.4	− 2.24	− 14.5	− 9.75
(iii) Source composition term (‰)	− 12.97	− 8.60	− 9.17	− 10.17	− 9.51	− 13.47	− 9.53	− 6.69	− 13.11	− 10.07
(Sum of i, ii,iii) × P_tag_/P_total_ (‰)	− 3	− 0.55	− 0.7	− 0.76	− 0.99	− 3.47	− 0.65	− 0.19	− 2.02	− 1.0
Simulated δ^18^O_ptag_ (‰)	− 2.87	− 0.52	− 0.75	− 0.73	− 1.11	− 3.02	− 0.53	− 0.09	− 1.69	− 0.95

Generally, the source composition is the dominant term determining the δ^18^O_ptag_ at the sink in the SW monsoon season for the major sources (Southern and Central Indian Ocean, Arabian Sea, and recycling). The source composition of the Southern and Central Indian oceans are ~ − 13‰ and − 10‰, respectively. The rainout effect (depletion of δ^18^O_vtag_ between the source and the sink due to condensation/rainouts) causes a depletion of ~ 3‰ each for the Southern and Central Indian oceans in the summer monsoon season. The condensation term represents the enrichment of heavier isotopes in precipitation relative to the vapor (as the heavier molecule condenses preferentially, precipitation is isotopically more enriched than the vapor), and is similar (~ + 5‰) for all the major sources. The δ^18^O_ptag_ values of the Arabian Sea and precipitation recycling in the SW monsoon season are relatively enriched compared to the Indian Ocean sources, due to their enriched source composition and smaller effects of rainouts. The Arabian sea is enriched due to its warm, tropical nature. The reason for enriched recycled vapor/precipitation compared to Indian Ocean sources is that recycled vapor (through evapotranspiration from soil water and plants) does not undergo fractionation during evapotranspiration, unlike the oceanic vapor sources^42,43^. Further, δ^18^O of the soil water reflects the δ^18^O_p_ and is more enriched than the ambient vapor^42^.

The δ^18^O_vtag_ and δ^18^O_ptag_ values of precipitation recycling in the NE monsoon season are similar to those in the SW monsoon season due to only minor depletion in source composition in the NE monsoon, and similar rainout and condensation terms in both seasons. An overly depleted source composition (~ − 13.5‰) and the large rainout term (~ − 11.6‰) for the North Pacific due to a larger distance from the sink, and smaller condensation enrichment than other sources, lead to a highly depleted δ^18^O_ptag_ for North Pacific. The rainout terms for the Southern and Central Indian oceans in the NE monsoon season increase by more than three-fold, although their source compositions remain similar in both seasons, leading to more negative δ^18^O_vtag_ values at the sink (Fig. 5). However, reduced precipitation from these sources limit their δ^18^O_ptag_ contribution to the NE monsoon season. The relatively enriched source vapor composition of the Bay of Bengal, together with a relatively high enrichment during condensation and a smaller rainout effect leads to a comparatively positive δ^18^O_ptag_ value for this source in the NE monsoon season. For the Arabian Sea, an increased condensation enrichment and reduced precipitation in the OND season lead to similar δ^18^O_ptag_ values in both seasons, despite a depletion in the source composition and a stronger rainout effect in the NE monsoon season leading to more depleted δ^18^O_vtag_ at the sink.

Hence, intense precipitation from the Indian Ocean sources and their depleted source compositions dominate the isotopic ratios of SW monsoon precipitation. Increased water vapor contribution from the relatively more depleted North Pacific source region (due to depleted source composition and rainout effect during transport) is the major cause of the more negative net δ^18^O_p_ in the NE monsoon season. Most of the major sources of NE monsoon precipitation (except for the precipitation recycling) have a larger rainout term due to the changes in circulation, which brings water vapor with relatively more depleted δ^18^O_vtag_ from these sources. However, the effect of several of these source regions (especially the Indian ocean sources and the Arabian Sea) on the net precipitation-weighted δ^18^O_p_ in the NE monsoon season is diminished due to their reduced contributions to precipitation. Increased condensation enrichment simulated for many sources in the NE monsoon season is likely due to reduced condensation, and post-condensation processes such as isotopic equilibration of the condensate with near-surface vapor and subsequent re-enrichment of precipitation, previously observed in light rains^44^, as the precipitation from the major sources except for the North Pacific in the NE monsoon season is light (0–1 mm day^−1^; Supplementary Fig. S6). Conversely, heavy precipitation is typically less equilibrated with the lower level vapor^44^, which likely contributes to relatively smaller condensation enrichment terms for many sources in the SW monsoon season and the North Pacific in the NE monsoon season. However, a comprehensive assessment of the effect of post-condensation processes is beyond the scope of this study. Such an assessment merits further studies in the future.

### The “tropical amount effect” in the two seasons

**Figure 6:**
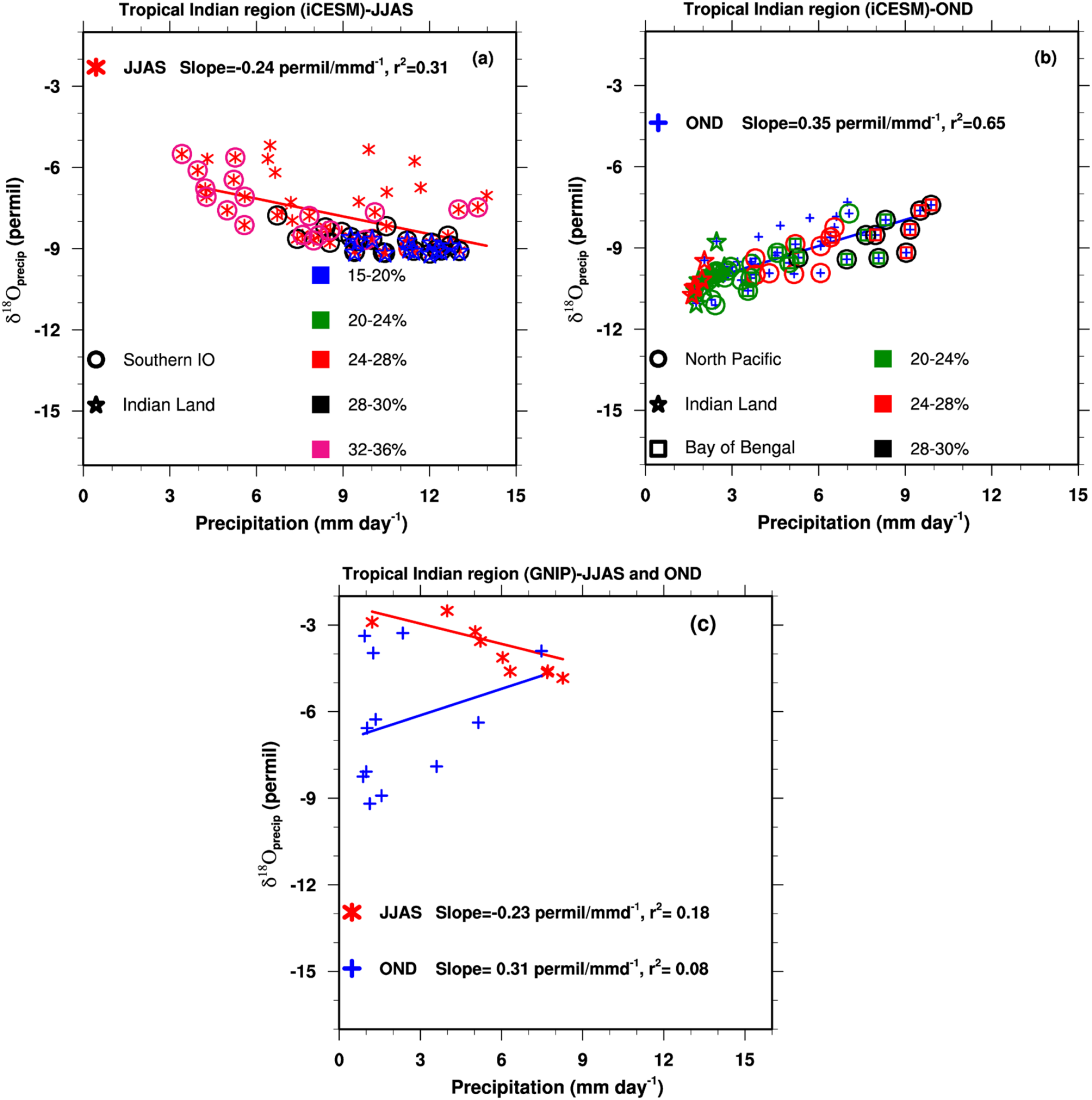
Linear regression between JJAS (red markers and line) and OND (blue markers and line) mean δ^18^O_p_ (in ‰) and precipitation (in the range of 0–14 mm day^−1^) over the tropical Indian land region (8° N–20° N, 65° E–88° E). Panel (**a**) Model-simulated JJAS values. Percentage contributions of the Southern Indian Ocean and Indian land recycling to the total precipitation are overlaid on the JJAS scatters. 15–20% contribution markers are shown only for precipitation recycling as its contribution is confined to this range. Panel (**b**) is similar to panel (**a**) but for the OND season, and percentage contributions of North Pacific, Indian land recycling, and the Bay of Bengal to the total precipitation are overlaid on the OND scatters. For example, a red square and black circle overlaid on the blue-cross OND scatter imply 24–28% of the contribution from the Bay of Bengal and 28–30% of the contribution from the North Pacific to the total precipitation. Panel (**c**) Regressions calculated from OND and JJAS data from GNIP stations in the geographical region. The spatial slopes and r^2^ from the regression analysis are shown in the panels. The figures were created using NCAR Command Language (NCL) Version 6.6.2 (http://www.ncl.ucar.edu/).

The amount effect^1^ is used widely in paleomonsoon reconstructions. We estimate the simulated and observed (precipitation and δ^18^O_p_ data from GNIP stations, see Methods) seasonal spatial amount effect over the tropical Indian land region (8° N–20° N, 65° E–88° E) as the linear regression between JJAS and OND mean precipitation and corresponding mean seasonal δ^18^O_p_ (Fig. 6). The simulated spatial slope of the regression analysis in the SW monsoon season is − 0.24‰/mm day^−1^ (r^2^ 0.31, for precipitation rates up to ~ 14 mm day^−1^). This moderate inverse relationship is consistent with that estimated from GNIP observations (− 0.23‰/mm day^−1^ (r^2^ 0.18; Fig. 6c), which suggests that the tropical δ^18^O_p_ in the SW monsoon season is influenced by the strength of local precipitation.

A separate regression analysis between the precipitation from major sources (P_tag_) and corresponding δ^18^O_ptag_ values over the tropical Indian region is shown in Supplementary Fig. S6. The regression analysis for several sources in both seasons shows smaller coefficients of determination. A distinct inverse relationship between δ^18^O_ptag_ and P_tag_ is seen only for the precipitation recycling in the SW monsoon season (− 0.4‰/mm day^−1^, r^2^ 0.75). The Indian Ocean sources, especially the Southern Indian Ocean, contribute highly to both high and low precipitation rates, and their δ^18^O_ptag_ values are similarly depleted irrespective of precipitation amount (Fig. 6a, Supplementary Fig. S6). The results suggest that the moderate amount effect simulated in the SW monsoon season is a result of a robust P_tag_–δ^18^O_ptag_ inverse relationship for the recycling as its contributions (~ 15–20%) are confined to large precipitation rates (Fig. 6a), along with a large supply of depleted precipitation from the Indian Ocean sources.

However, the amount effect is absent in the NE monsoon precipitation in both model simulations and observations (Fig. 6b), and regression analysis for the model results captures a positive slope with a higher goodness-of-fit compared to the SW monsoon season (0.35‰/mm day^−1^, r^2^ 0.65). The slope for the observations in the OND season is uncertain (+ 0.31‰/mm day^−1^, r^2^ 0.08), possibly because of sparse station data.

The simulated low precipitation rates (≤ 4 mm/day) in the OND season in the tropical Indian region are more depleted compared to high precipitation, as the low precipitation is dominated by contributions from relatively depleted North Pacific and precipitation recycling sources (> 50% together, Fig. 6b), the latter contributing majorly to low precipitation. The high precipitation rates (≥ 7 mm/day) are comparatively enriched and are dominated by the North Pacific and the Bay of Bengal sources. The Bay of Bengal is a highly enriched source (Supplementary Fig. S6) and likely leads to enriched higher precipitation, despite depleted contributions from the North Pacific. Further, except for the North Pacific with a noticeable inverse P_tag_–δ^18^O_ptag_ relationship (− 0.5‰/mm day^−1^, r^2^ 0.56; Supplementary Fig. S6), positive slopes of regression are estimated for several sources in the NE monsoon season, the reason for which is unclear from our results. Factors such as reduced condensation, the colder temperature in the season, and increased condensation enrichment (Table 1) are likely to have contributed to this. Hence, the positive slope of regression in the NE monsoon season (Fig. 6b) is likely due to concerted contributions of the depleted major sources to the low precipitation, the larger contribution of enriched Bay of Bengal water vapor to the high precipitation, and several sources with a positive P_tag_–δ^18^O_ptag_ relationship.

The strong positive relationship in the NE monsoon season dominates the mean annual P–δ^18^O_p_ relationship in the tropical Indian region (0.52/mm day^−1^, r^2^ = 40, Supplementary Fig. S7a). The regression relation estimated from the GNIP observations for the mean annual values (− 0.05‰/mm day^−1^, r^2^ 0.02; Supplementary Fig. S7c) is not robust, likely due to inadequate data, and hence we cannot unambiguously substantiate our results. However, a moderate annual spatial amount effect is estimated from the model results and GNIP observations in the whole Indian domain (8° N–30° N; Supplementary Fig. S7b,d), which includes regions where the influence of NE monsoon precipitation is weak. The precipitation-δ^18^O_p_ regression relationship from the mean JJAS observations in the Indian domain (− 0.63‰/mm day^−1^, r^2^ 0.47; Supplementary Fig. S7d) is also stronger than in the tropical Indian region. Therefore, we suggest the annual amount effect may prevail in regions where the influence of NE monsoon precipitation is low, and these findings have implications for the interpretation of water isotope variations in climate proxies. However, a larger network of observational data is required to draw robust conclusions on data-model comparisons.

## Discussions

We have analyzed the major water vapor sources for the precipitation in the Indian region for the SW and NE monsoon seasons using the isotope-enabled Earth System Model iCESM1. Further, we studied the effects of the water vapor sources on the δ^18^O_p_, which have implications for paleoclimate reconstructions. The iCESM1 is able to simulate the seasonal changes in the sources of water vapor in the Indian region and their effects on water isotopes in precipitation. The results show that the Indian Ocean sources, Arabian Sea, and continental recycling are the major sources of SW monsoon precipitation in the Indian domain, in agreement with previous studies using Lagrangian techniques^21–25^. The substantial contribution of the North Pacific to the NE monsoon precipitation is important, as observations show that positive phases of El Niño–Southern Oscillation (ENSO) are favorable for stronger NE monsoon^16^, and that excess NE monsoon years are associated with more water vapor derived from the western Pacific Ocean^37^.

Similar to the contributions to net precipitation, the Indian Ocean sources together (South and Central) are the largest contributors to the net precipitation-weighted δ^18^O_p_ in the Indian domain in the SW monsoon season. This is because the Indian ocean sources have a relatively depleted source composition, and they make the largest (~ 38%) contribution to the summer precipitation. We explain the differences in δ^18^O_p_ values in the NE monsoon season in the Indian domain relative to the SW monsoon season as predominantly driven by changes in circulation and water vapor sources. The principal reason behind the more depleted δ^18^O_p_ values in the NE monsoon precipitation is the increased advection of depleted water vapor from the North Pacific Ocean and its considerable contribution to the net precipitation. Arguably, a significant change in water vapor source location due to any circulation change in the past can alter the interpretations of the δ^18^O signal in the climate proxy records. Hence, we suggest that such changes in the water vapor sources in the past need to be understood better using water vapor and isotope tracking Earth system models.

The results suggest a moderate amount effect in the SW monsoon season, and the annual amount effect is likely to prevail in regions where the influence of NE monsoon precipitation is low. Hence, we suggest oxygen isotope data from proxy records in the Indian domain can help reconstruct the past summer monsoon precipitation variability. However, the moderate relationship also suggests the influence of factors other than the amount of precipitation on the δ^18^O_p_, such as the effects of water vapor sources, circulation, and types^4,41,45^ of precipitation. Previous studies have found that El Niño–Southern Oscillation (ENSO) and Indian Ocean Dipole (IOD) influence the interannual variability of Indian monsoon precipitation^16,17,46,47^. Distinguishing the characteristics of major water vapor sources under the influence of ENSO/IOD will be highly beneficial for understanding the Indian monsoon variability. However, the prescribed SST simulation does not capture the observed ENSO/IOD teleconnections with the Indian monsoon precipitation (Supplementary Fig. S8 and Supplementary Text S1), as also documented in previous studies using CESM^48^. Hence, we suggest that future work concerning interannual variability of source contributions should include fully-coupled simulations of iCESM. Weather phenomena such as monsoon low-pressure systems^49,50^ and tropical cyclones^51^ likely affect the sources of water vapor for Indian precipitation. However, as the average lifetime of these weather systems is less than a week, an investigation into their effects would require analysing data at high temporal resolution (e.g. daily data). Since we have focused on long-term seasonal climatology, the effects of such weather phenomena on the water vapor sources have not been investigated in this paper. Such an investigation merits further studies.

In summary, the present study exemplifies the use of water tagging methods in an Earth system model to identify the major sources of precipitation, and their effects on water isotope ratios of monsoon precipitation, with implications for future climatic studies and past climate reconstructions.

## Methods

### Model and experimental setup

We use the National Center for Atmospheric Research (NCAR)’s Community Earth System Model (CESM) version 1.2 equipped with water-isotope tracers (iCESM1^29^) for the simulation. iCESM1 can transport water isotope tracers in the atmosphere, land, ocean, sea ice, and river runoff components. The water vapor tracking capabilities of iCESM1 have been used in a few recent studies to examine the orbital-scale variability in δ^18^O_p_ in South Asia^7^ and to evaluate the importance of water vapor source locations for the Chinese speleothem records^8,30^. We employ the prescribed ocean mode for our simulation, where the atmosphere and land are dynamically coupled, and sea-surface temperature (SST)/sea-ice components are prescribed. The atmosphere and land models respectively are the isotope-enabled versions of the Community Atmosphere Model version 5 (iCAM5^40^), and the Community Land Model version 4 (iCLM4^52^). Isotopic fractionation processes are included in iCAM5’s physical parameterizations, and the fractionation coefficients are taken from empirically derived formulas^53–55^. The iCLM4 model includes water isotope hydrology in parallel to that of CLM4^52^ and is a major upgrade from the bucket models that have been used to represent isotope exchanges from the land in the previous generation of isotope-climate models^56^. A novel feature of iCESM is water-tagging, whereby the model can track the water vapor and water isotope species from different source regions by tracking the evaporation from each source. The water tagging feature in the iCESM has been used in several recent studies^7,8,57–59^. We set the iCESM1 to run in an Atmospheric Model Intercomparison Project (AMIP) configuration (prescribed sea surface temperatures and sea ice concentrations). The simulation is forced with the Hadley Centre for Climate Prediction and Research sea ice and Sea Surface Temperatures (HadISST^60^) from the years 1979 to 2003 (Supplementary Fig. S1). We use a monthly-varying present-day data set of sea surface water isotope ratios for the simulation. This data was prepared by merging observed long-term annual water isotope ratios^61^ with simulated long-term monthly ocean surface water isotope data for the present day^29^.

### Global network of isotopes in precipitation (GNIP) data

We selected observed precipitation and δ^18^O_p_ data (available at https://nucleus.iaea.org/wiser/index.aspx as long-term monthly mean data) from all the available Global Network of Isotopes in Precipitation (GNIP) stations from the Southeast Asian region (details of the stations in India are given in Supplementary Table S2). We calculated the JJAS, OND, and annual means of precipitation and δ^18^O_p_ data in the Indian domain irrespective of the length of the available data, as many stations in India have only a few years or few months of measurements. The annual means are calculated if the station has at least 9 long-term mean monthly values of precipitation and δ^18^O_p_, as the majority of the stations do not have year-round measurements. The stations in the tropical Indian land region (8° N–20° N, 65° E–88° E) are used for the calculation of the seasonal spatial amount effect, shown in Fig. 6.

### Tagged source regions

**Figure 7:**
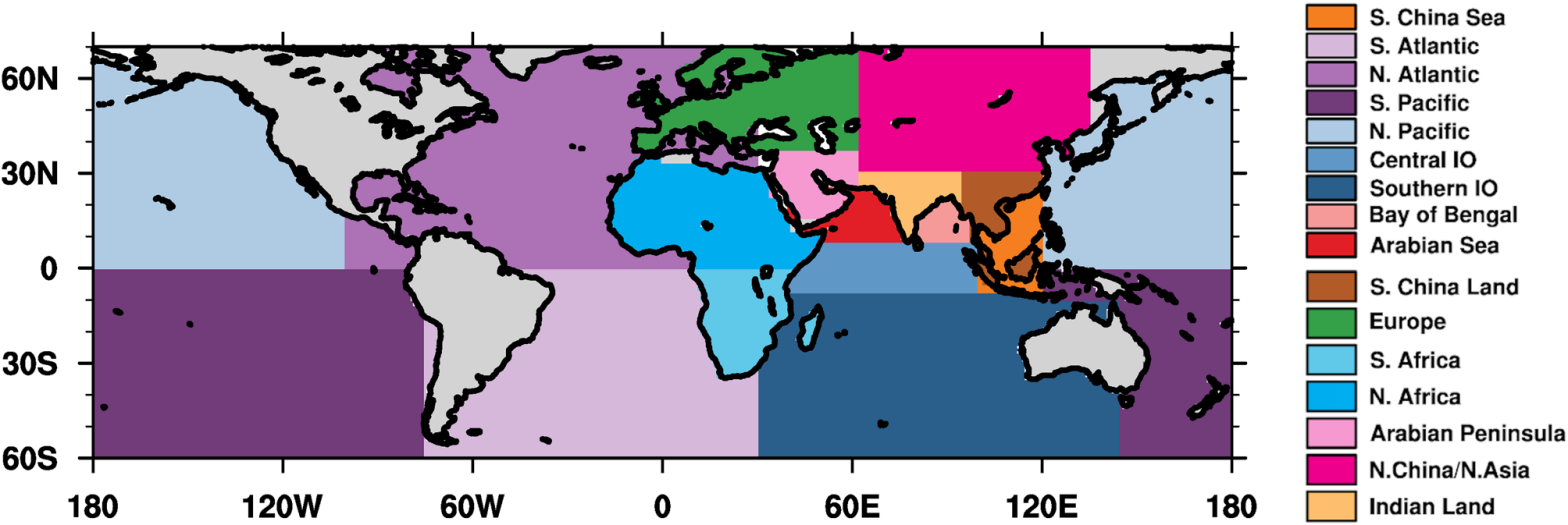
Tagged water vapor source regions for precipitation over the Indian land region. Grey regions on the map are not tagged. The source “Indian land” denotes precipitation recycling from the Indian land region. The figure was created using NCAR Command Language (NCL) Version 6.6.2 (http://www.ncl.ucar.edu/).

The evaporative fluxes of the oxygen isotope species and water vapor from 16 oceanic and terrestrial regions (Fig. 7, Supplementary Table S1) around the Indian subcontinent, including continental India (to calculate the contribution of precipitation recycling) are tracked. The source regions have been selected based on existing studies on major sources of water vapor in India^22,24^. The source regions are exclusively either land or ocean, selected using land and ocean fractions in the surface data used in the model.

We calculate the major sources of water vapor to India for the SW summer monsoon (JJAS) season and NE winter monsoon season (OND) for the mean of the years 1980–2003.

The simulated climatological precipitation (seasonal and annual) and circulation over India are compared with precipitation data from the Global Precipitation Climatology Project (GPCP^31^) and wind data from ERA5 reanalysis^32^. The observed present-day isotope ratios in precipitation are taken from the GNIP database^39^.

### Decomposition of δ^18^O_ptag_

The δ^18^O_ptag_ of a particular water source at the sink region could be estimated as the sum of three terms, (i) the δ^18^O of water vapor at the source region, (ii) the effect of rainouts on the δ^18^O of the tagged vapor, and (iii) the enrichment of δ^18^O_ptag_ during condensation from the ambient tagged vapor^7^. Using this, we estimate the dominant effects (condensation, rainouts, source composition) that determine the δ^18^O_ptag_ of major source tags in the two seasons. The three terms are defined as

#### Condensation term

$${\delta }^{18}{{O}_{condensation}}_{\left[tag\right]}={\left({\delta }^{18}{O}_{Psink}-{\delta }^{18}{O}_{wvsink}\right)}_{season}\times {\left(\frac{{P}_{tag}}{{P}_{total}}\right)}_{season}.$$

#### Rainout term

$${\delta }^{18}{O}_{Rainout\left[tag\right]}={\left({\delta }^{18}{O}_{wvsink}-{\delta }^{18}{O}_{wvsource}\right)}_{season}\times {\left(\frac{{P}_{tag}}{{P}_{total}}\right)}_{season}.$$

#### Source composition term

$${\delta }^{18}{O}_{Source\left[tag\right]}={\left({\delta }^{18}{O}_{wvsource}\right)}_{season}\times {\left(\frac{{P}_{tag}}{{P}_{total}}\right)}_{season}.$$

δ^18^O_wvsource_ and δ^18^O_wvsink_ are the isotope ratios of water vapor at 850 hPa of each tag at their respective source regions and at the Indian sink, respectively.

We note the existing framework^7^ to derive the contributions of condensation, rainout, source changes, and source composition to the changes in δ^18^O_p_ between two climate states. However, we do not use this framework in our study to differentiate the δ^18^O_p_ between the SW and NE seasons, as the framework assumes a small surface temperature change between the climate states. The large surface temperature difference between the SW and NE monsoon seasons (Supplementary Figs. S1, S2) can cause errors in the estimates due to the effects of temperature change on the fractionation process and may introduce large residuals.

## Supplementary Information

Supplementary Information.

## Data Availability

The data from the simulation used in the study is available at https://doi.org/10.5281/zenodo.6856201. The GNIP station data is accessible at https://nucleus.iaea.org/wiser/index.aspx. The GPCP data is available at https://psl.noaa.gov/data/gridded/data.gpcp.html. The ERA5 data is available for download at https://www.ecmwf.int/en/forecasts/datasets/reanalysis-datasets/era5.
