# Supplementary material for: Sources of water vapor and their effects on water isotopes in precipitation in the Indian monsoon region: a model-based assessment
Source: Sci Rep. 2023 Jan 13;13:708. doi: 10.1038/s41598-023-27905-9 (PMC9839761; doi:10.1038/s41598-023-27905-9)
Supplement: Supplementary file 1 — Supplementary Information. [file 41598_2023_27905_MOESM1_ESM.pdf]

Supplement to “Sources of Water Vapor and their Effects on Water Isotopes in Precipitation in the Indian Monsoon Region: A Model-Based Assessment”

Thejna Tharammal[1,*], Govindasamy Bala[1], and Jesse M Nusbaumer[2]

[1]Centre for Atmospheric And Oceanic Sciences, Indian Institute of Science, Bangalore, India.
[2]National Center for Atmospheric Research, Boulder, Colorado, United States.

Corresponding author: Thejna Tharammal (thejnat@iisc.ac.in, thejna@gmail.com)
*Currently at Interdisciplinary Centre for Water Research, Indian Institute of Science, Bangalore, India.

Contents:

- Supplementary text S1: Relationship between simulated interannual variability of monsoon precipitation and ENSO and IOD indices
- Supplementary Figures S1 to S8
- Supplementary Table S1: Geographical coordinates of the 16 source regions.
- Supplementary Table S2: Details on GNIP stations in the Indian region

Supplementary Text S1: Relationship between simulated interannual variability of monsoon precipitation and ENSO and IOD indices

The El Niño–Southern Oscillation (ENSO) is natural climate variability related to tropical Pacific SST changes and is a major forcing for the interannual variability of the Indian monsoon[1]. Further, the Indian Ocean Dipole (IOD[2]) is another key factor in the interannual variability in the tropical Indian Ocean SST and it influences the Indian monsoon precipitation. Observations and modeling studies[1,3–5] show links between the strength of the southwest summer monsoon (SW) precipitation and ENSO. Generally, the positive phase of ENSO (El-Niño) is associated with reduced SW monsoon precipitation, and the negative phase (La-Niña) is associated with above-normal precipitation in the Indian region[6]. A positive (negative) phase of IOD is associated with an increased (decreased) SW monsoon precipitation over India. Similarly, previous studies find that positive phases of ENSO and IOD are associated with above-normal precipitation in the northeast winter monsoon (NE) season[7–10]. Hence, in this text, we assess the relationship of the simulated interannual variability in the SW and NE monsoon precipitations with the ENSO and IOD indices.

Methods: The characteristics of the ENSO events are classified using indices that estimate the anomalies in equatorial Pacific SSTs. We use the Oceanic Niño Index to estimate the interannual ENSO variability using the HadISST SST dataset used to force the iCESM1 simulation. The Oceanic Niño Index (ONI) is estimated (for the JJAS mean and OND mean) during the years 1980-2003 as the 3-month running domain mean SST anomaly (difference from the mean) in the Nino 3.4 region (located in the central Pacific Ocean, 170°W -120°W; 5°S -5°N). If the ONI index is greater than +0.5 C, we classify it as El Niño, and if the ONI is less than -0.5, it is classified as La Niña. The intensity of the Indian Ocean Dipole (for the JJAS and OND seasons) is measured using the HadISST SST dataset by a commonly used index (referred to as the Dipole Mode Index, DMI), which is the difference between SST anomalies in two regions of the tropical Indian Ocean, West (10°S to 10°N; 50°E to 70°E) and East (10°S to 0°S; 90°E to 110°E). DMI index values of >0.4 °C are a positive IOD, and values below −0.4 °C are considered negative IOD.

Results and discussion: The regression analysis (supplementary Fig. S8) between ENSO/IOD indices with the strength of SW and NE monsoon precipitation over the years 1980-2003 shows that the fixed-SST simulation that we carried out is incapable of simulating

the observed negative relationship between SW monsoon precipitation and the ENSO index, and it also fails to simulate a robust positive relationship between the IOD index and SW monsoon precipitation. The model also does not simulate the positive relationship between the ENSO index and the NE monsoon precipitation reported in observational studies[7]. Further, simulated NE monsoon precipitation has an inverse relationship with the IOD index (supplementary Fig. S8), opposite in sign of the observations[7]. The inability of the NCAR atmosphere model to simulate the interannual variability and the ENSO-Indian monsoon relationships has been discussed in previous studies[11]. They assess a lack of atmosphere-ocean coupling limits the model's ability to reliably simulate the interannual variability of ISM and its relationships with the IOD and ENSO events, which are better resolved in the coupled atmosphere-ocean simulations using the CESM.

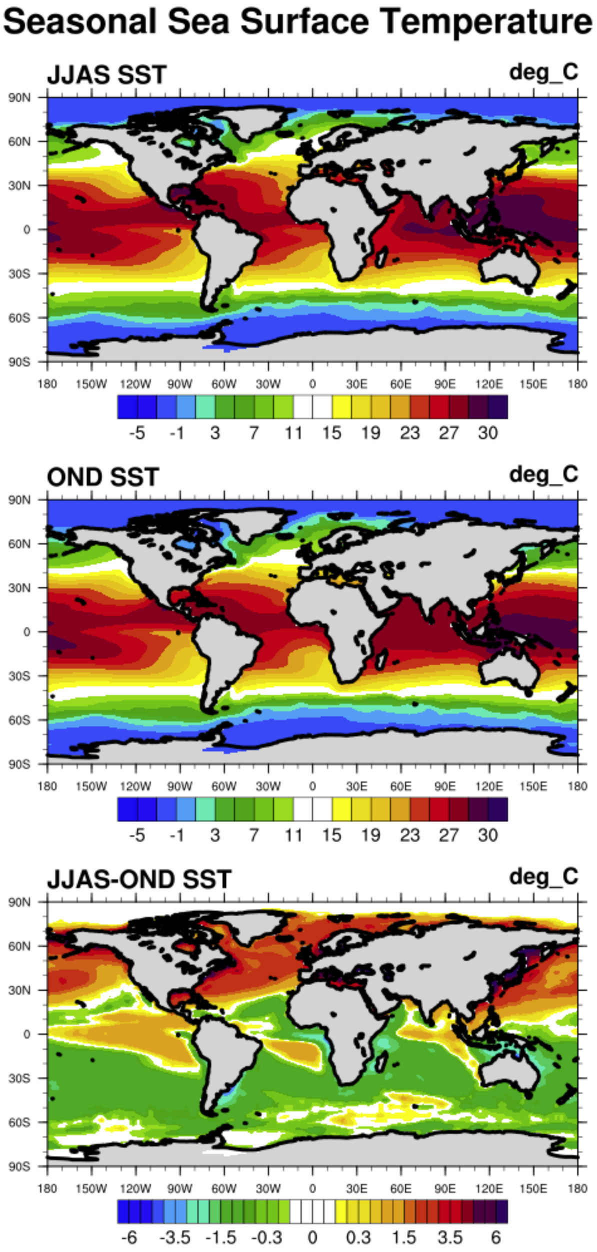

Supplementary Fig. S1: The climatological seasonal (JJAS and OND; years 1979-2003) means of sea surface temperature -SST (in degrees Celsius) and their difference. Data is from Hadley Centre for Climate Prediction and Research sea ice and Sea Surface Temperatures (HadISST) for the years 1979-2003 and is used to force the model simulation. The figures were created using NCAR Command Language (NCL) Version 6.6.2 (http://www.ncl.ucar.edu/).

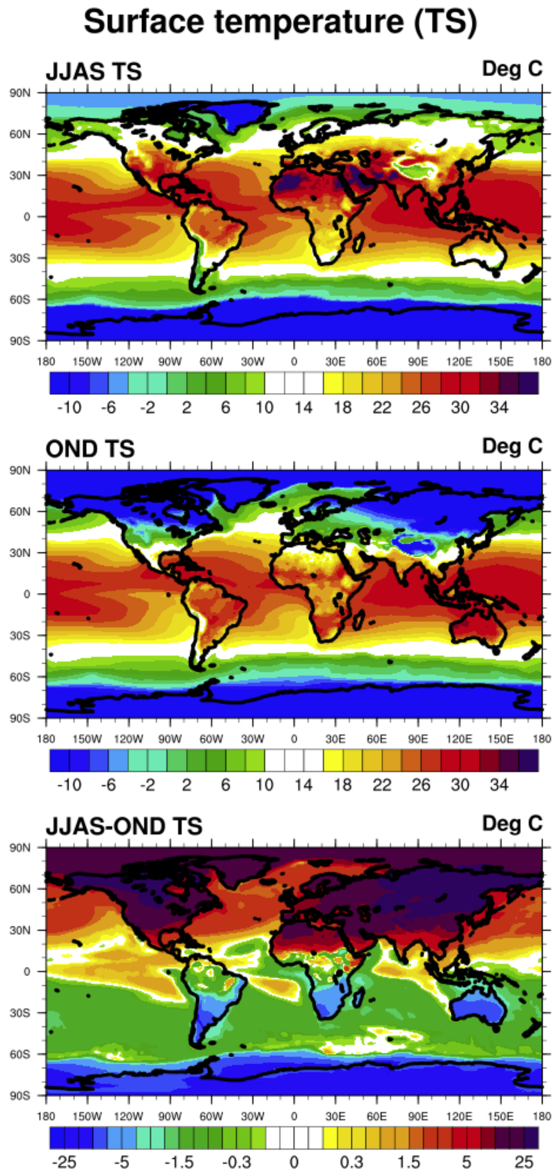

Supplementary Fig. S2: The simulated climatological seasonal (JJAS and OND; years 1979-2003) means of surface temperature (in degrees Celsius) and their difference. The figures were created using NCAR Command Language (NCL) Version 6.6.2 (http://www.ncl.ucar.edu/).

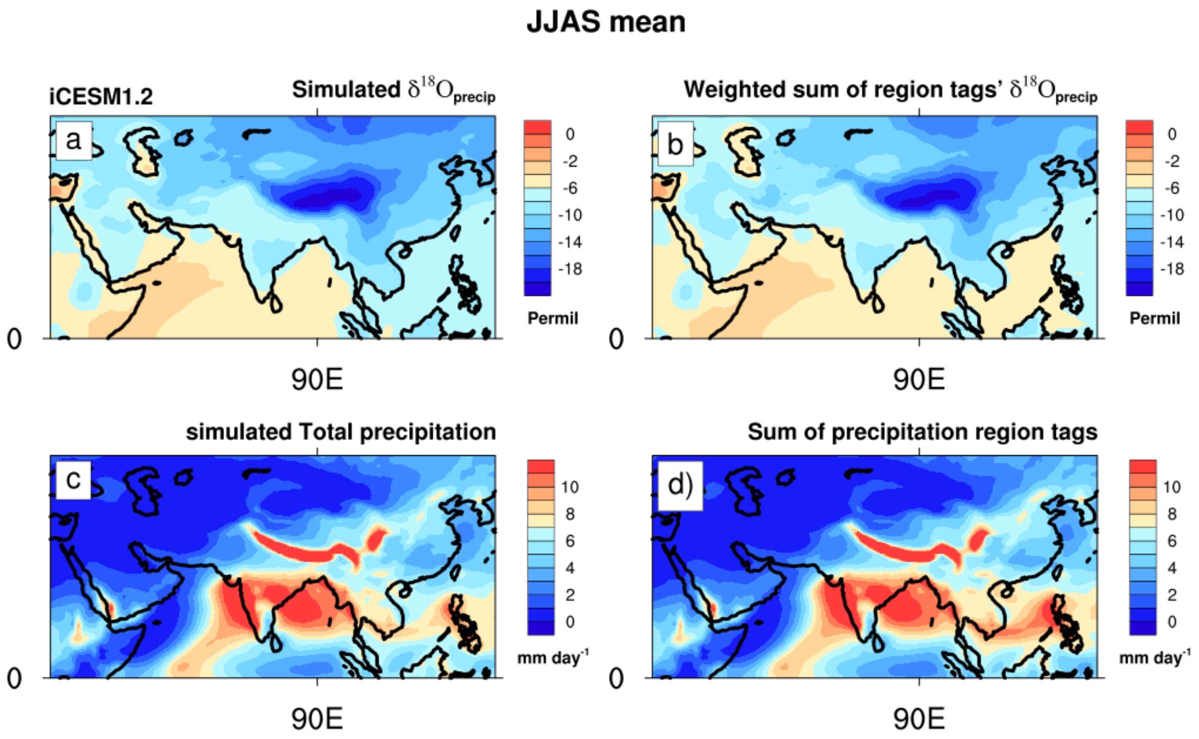

Supplementary Fig. S3: a) The simulated mean $\delta^{18}O_{precip}$ in the JJAS season, and b) precipitation weighted sum of mean $\delta^{18}O_{ptag}$ from the 16 tagged regions. c) simulated mean total precipitation in the JJAS season, d) sum of the precipitation contribution from the 16 tagged regions. The figures were created using NCAR Command Language (NCL) Version 6.6.2 (http://www.ncl.ucar.edu/).

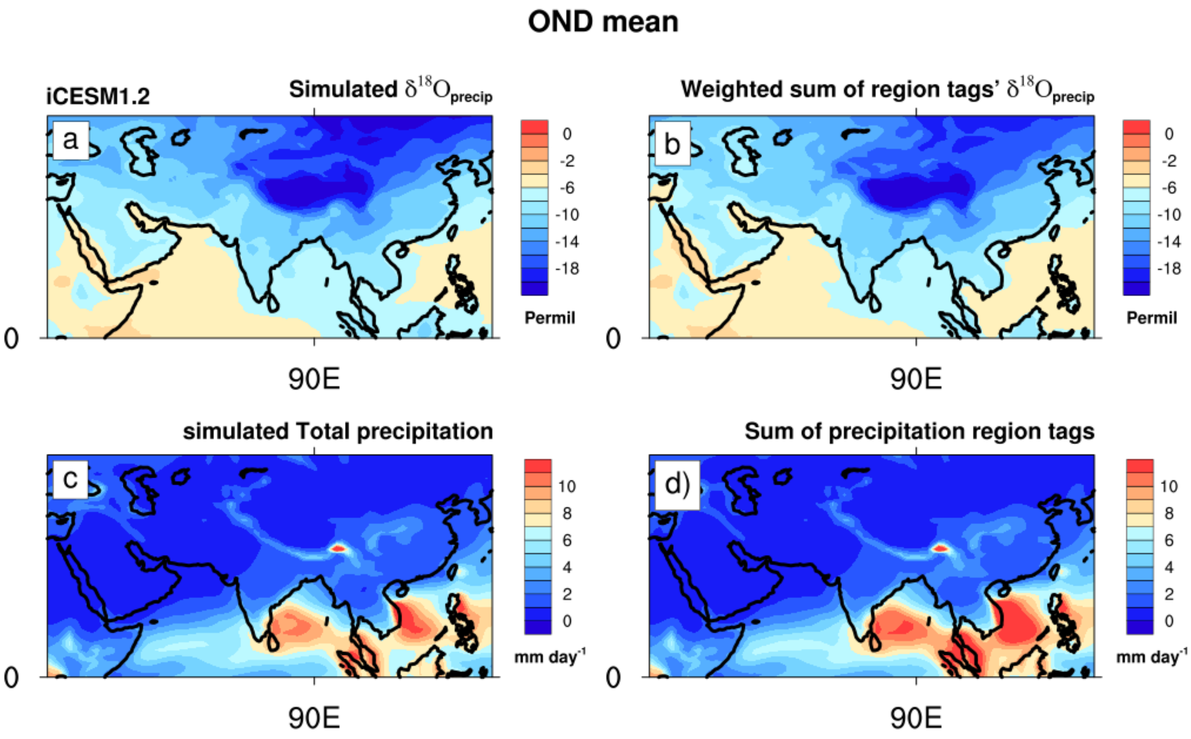

Supplementary Fig. S4: Same as S3, but for the OND season. The figures were created using NCAR Command Language (NCL) Version 6.6.2 (http://www.ncl.ucar.edu/).

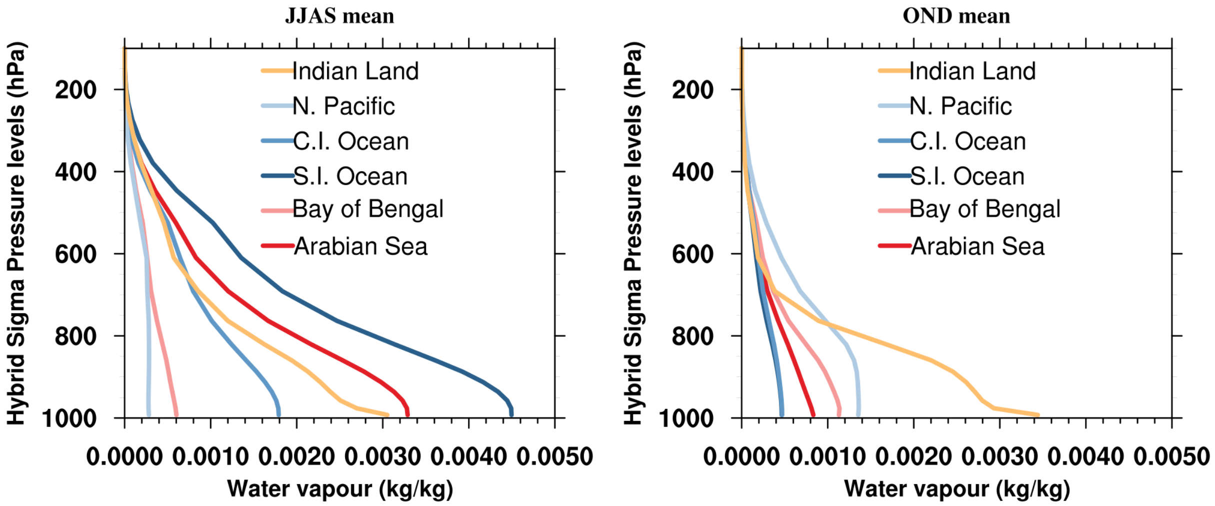

Supplementary Fig. S5: Vertical profiles of water vapor (averaged over the Indian domain) in kg/kg, from six major source regions for (left) JJAS and (right) OND seasons. These major sources are the Arabian Sea, Bay of Bengal, Southern Indian Ocean (S.I. Ocean), Central Indian Ocean (C.I. Ocean), North Pacific, and Indian land recycling (Indian Land). The figures were created using NCAR Command Language (NCL) Version 6.6.2 (http://www.ncl.ucar.edu/).

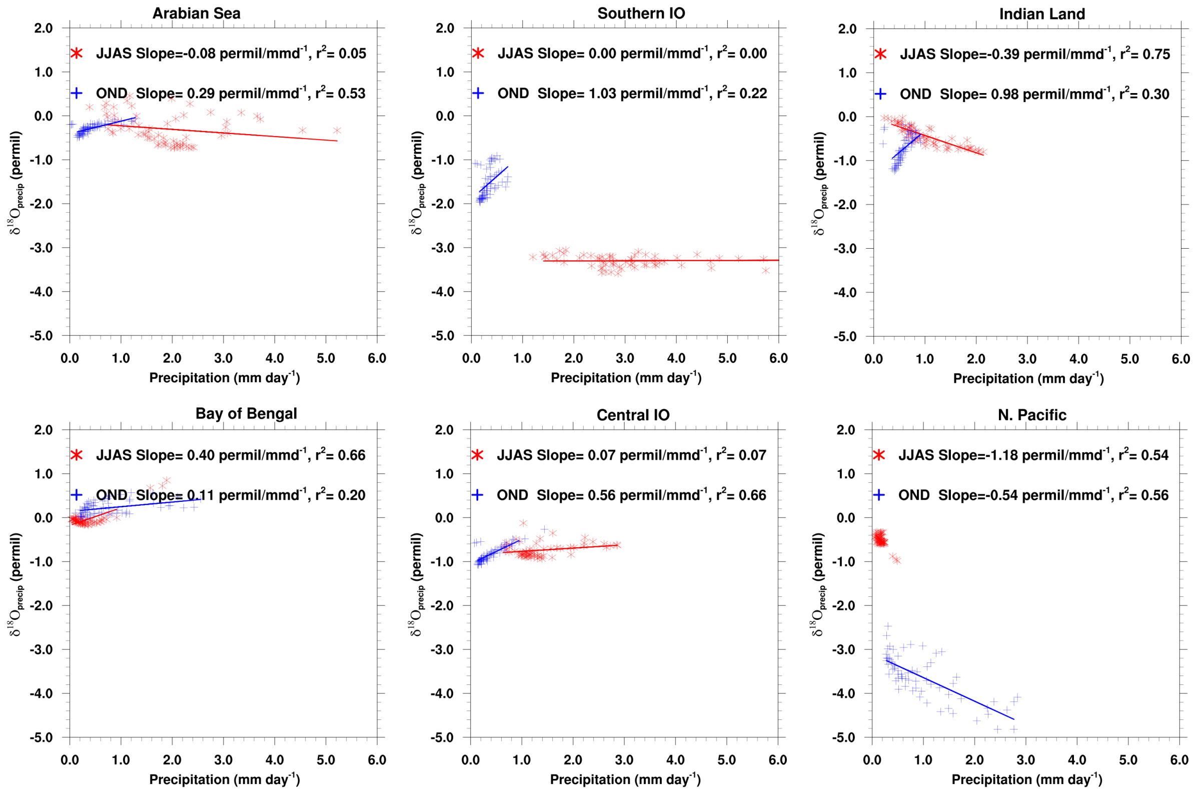

Supplementary Fig. S6: Linear regression between JJAS (red markers and line) and OND (blue markers and line) mean precipitation weighted $\delta^{18}O_{ptag}$ (in permil) from the six major sources and precipitation (in mm day^{-1}) from the respective sources over the tropical (8°N-20°N) part of the Indian domain. The regression, spatial slope, and r^2 from the regression analysis are shown in the figure. The figures were created using NCAR Command Language (NCL) Version 6.6.2 (http://www.ncl.ucar.edu/).

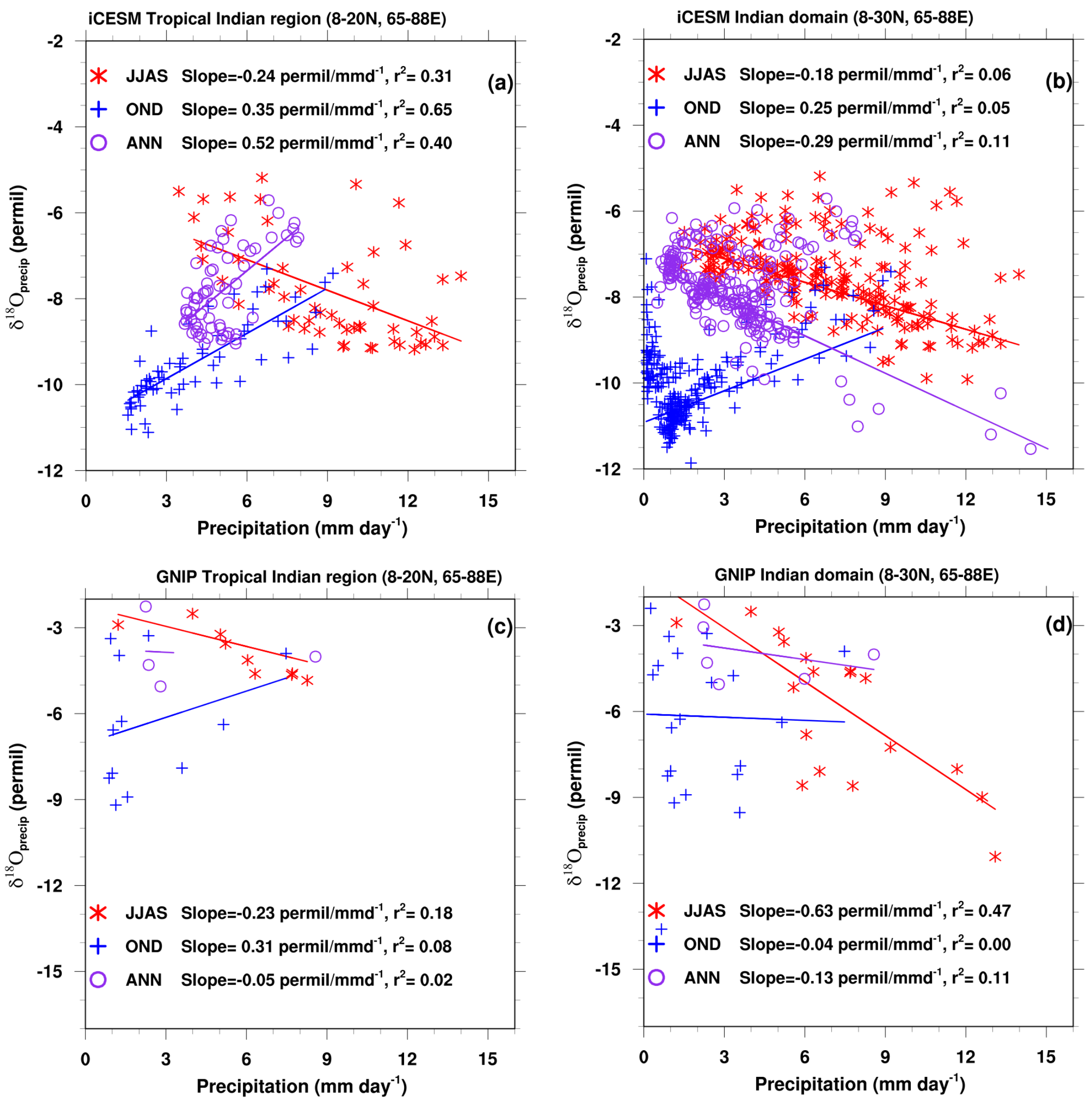

Supplementary Fig. S7: Linear regression between JJAS (red markers and line), OND (blue markers and line), and mean Annual (purple markers and line) mean precipitation weighted $\delta^{18}O_{ptag}$ (in permil) and precipitation (in mm day^{-1}) for iCESM simulated values (panels a and b) and GNIP station data (panels c and d). Panels a and c) show tropical (8°N-20°N, 65°E-88°E) part of the Indian domain, Panels b and d) show whole Indian domain (8°N-30°N, 65°E-88°E). The regression, spatial slope, and r^2 from the regression analysis are shown in the figure. The JJAS and OND scatter plotted in Panel (a) are the same as in Fig. 6a and 6b. The figures were created using NCAR Command Language (NCL) Version 6.6.2 (http://www.ncl.ucar.edu/).

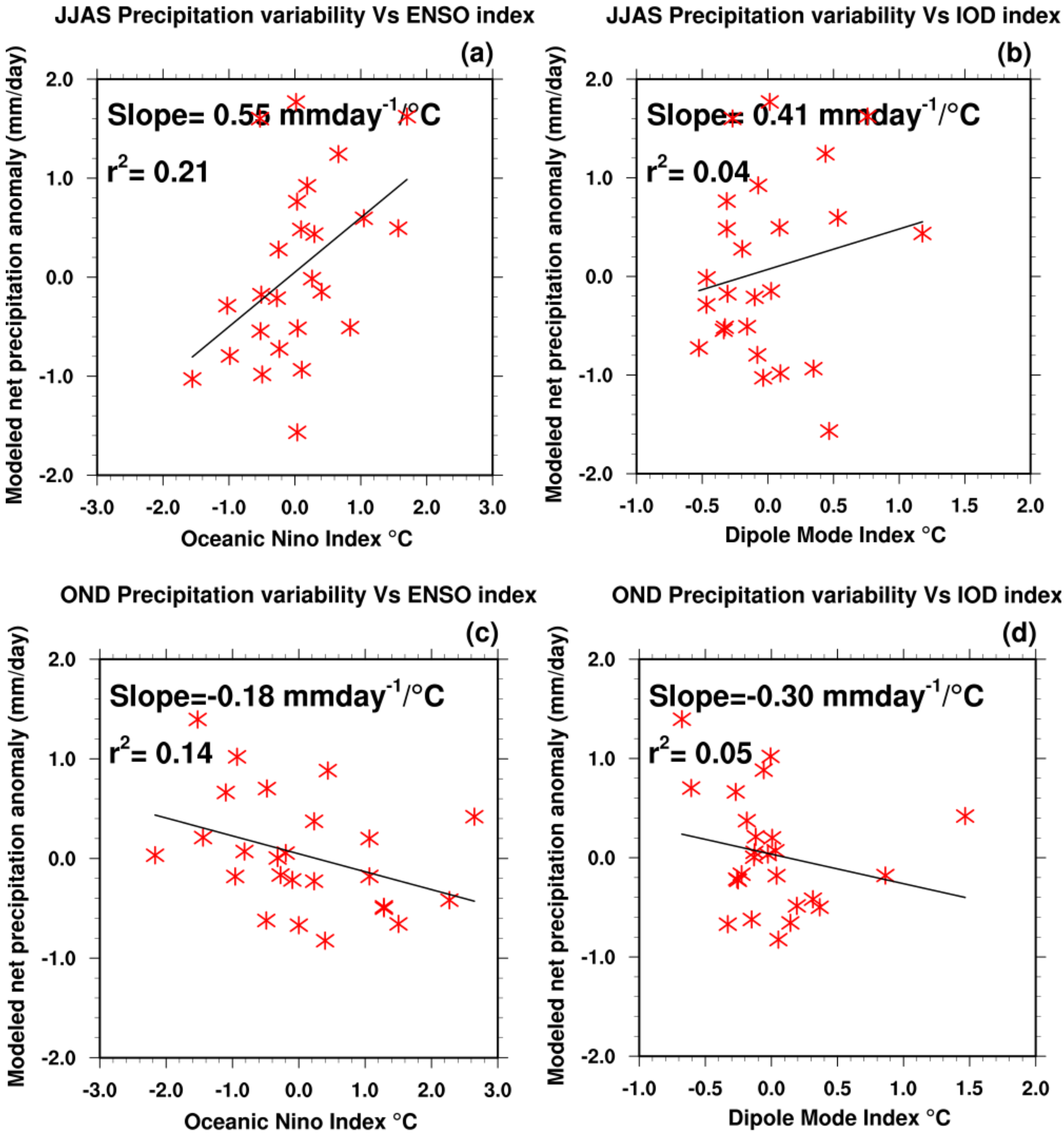

Supplementary Fig. S8: Relationship of interannual variability (difference from the mean over the years) of simulated SW and NE monsoon precipitation for the years 1980-2003 averaged over the Indian region with the Oceanic Nino Index (ONI in degree C) calculated from the HADSST forcing data used in the simulation (panels a and c for the SW and NE monsoons, respectively), and the Indian Ocean Dipole index (panels b and d for the SW and NE monsoons, respectively). The figures were created using NCAR Command Language (NCL) Version 6.6.2 (http://www.ncl.ucar.edu/).

Supplementary Table S1: Geographical coordinates of the 16 source regions. Note that the source regions are exclusively either land or ocean, selected using land and ocean fractions (from the surface data used in the model) inside the coordinates.

Tagged source region	Latitude range	Longitude range
The Arabian Sea	8°N-25°N	40°E-78°E
Bay of Bengal	8°N-24°N	78°E-100°E
South Indian Ocean	60°S -8°S	30°E-145°E
Central Indian Ocean	8°S-8°N	35°E-100°E
North Pacific Ocean	0°-65°N	90°W-120°E
South Pacific Ocean	60°S-0°	77°W-145°E
North Atlantic Ocean	0°-70°N	100°W-30°E
South Atlantic Ocean	60S°-0°	67°W-28°E
South China Sea	8°S-25°N	100°E-120°E
Indian land (Recycling)	5°N-30°N	62°E-95°E
N.China and N.Asia	30°N-70°N	62°E-135°E
Arabian Peninsula	15°N-40°N	33°E-62°E
Northern Africa	0°N-35°N	17°W-50°E
Southern Africa	35°S-0°N	11°W-50°E
Europe	37°N-70°N	13°W-62°E
South China land region	10°S-30°N	95°E-120°E

Supplementary Table S2: GNIP stations in the Indian region. Precipitation and water isotope data (available at WISER as long-term monthly mean) from all the stations were downloaded and processed to calculate the JJAS, OND, and annual means, irrespective of the length of the available data. The stations in the 8°N-20°N, 65°E-88°E are used for the calculation of tropical Indian spatial amount effect, shown in Fig. 6

SI. No	Station Name	Latitude	Longitude	Availability of mean annual values (Mean of minimum 9 months)
1	ALLAHABAD	25.45	81.73	No
2	BANGLORE	12.97	77.59	Yes
3	BELGAUM	15.88	74.49	No
4	BHOPALPALLI	18.27	79.52	No
5	CHINPAK	18.28	79.44	No
6	DEVPRAYAG	30.14	78.59	No
7	GANGOTRI	30.99	78.94	No
8	GOMUKH	30.92	78.94	No
9	GUWAHATI	26.19	91.79	Yes
10	HYDERABAD	17.45	78.47	Yes
11	KAKINADA	17.02	82.25	Yes
12	KAMALAPUR	18.29	79.54	No
13	KOLKATA	22.79	88.37	No

14	KOZHIKODE	11.25	75.78	Yes
15	LUCKNOW	26.87	80.93	No
16	MANERI	30.74	78.44	Yes
17	MUMBAI	18.9	72.82	No
18	NAINITAL	29.4	79.46	No
19	NASARAM	18.26	79.47	No
20	NEW DELHI	28.58	77.2	Yes
21	PATNA	25.57	85.07	No
22	RISHIKESH	30.112	78.3	No
23	ROORKEE	29.86	77.89	No
24	SAGAR	23.82	78.76	No
25	SHILLONG	25.57	91.88	Yes
26	TEHRI	30.35	78.48	Yes
27	TIRUNELVELI	8.72	77.71	No
28	TUNDLA BUZURG	18.32	79.47	No
29	UTTARKASHI	30.72	78.44	Yes

Supplementary References

1. Webster, P. J. & Palmer, T. N. The past and the future of El Niño. *Nature* **390**, 562–564 (1997).
2. Saji, N. H., Goswami, B. N., Vinayachandran, P. N. & Yamagata, T. A dipole mode in the tropical Indian Ocean. *Nature* **401**, 360–363 (1999).
3. Kanamitsu, M. & Krishnamurti, T. N. Northern Summer Tropical Circulations During Drought and Normal Rainfall Months. *Mon. Weather Rev.* **106**, 331–347 (1978).
4. Krishnamurti, T. N., Bedi, H. S. & Subramaniam, M. The Summer Monsoon of 1987. *J. Clim.* **2**, 321–340 (1989).
5. Chowdary, J. S. *et al.* Interdecadal Variations in ENSO Teleconnection to the Indo–Western Pacific for 1870–2007. *J. Clim.* **25**, 1722–1744 (2012).
6. Hrudya, P. H., Varikoden, H. & Vishnu, R. A review on the Indian summer monsoon rainfall, variability and its association with ENSO and IOD. *Meteorol. Atmos. Phys.* **133**, 1–14 (2021).
7. Rajeevan, M., Unnikrishnan, C. K., Bhate, J., Niranjan Kumar, K. & Sreekala, P. P. Northeast monsoon over India: variability and prediction. *Meteorol. Appl.* **19**, 226–236 (2012).
8. Yadav, R. K. Why is ENSO influencing Indian northeast monsoon in the recent decades? *International Journal of Climatology* vol. 32 2163–2180 (2012).
9. Sreekala, P. P., Vijaya Bhaskara Rao, S. & Rajeevan, M. Northeast monsoon rainfall variability over south peninsular India and its teleconnections. *Theoretical and Applied Climatology* vol. 108 73–83 (2012).
10. Sreekala, P. P., Rao, S. V. B., Rajeevan, K. & Arunachalam, M. S. Combined effect of MJO, ENSO and IOD on the intraseasonal variability of northeast monsoon rainfall over south peninsular India. *Clim. Dyn.* **51**, 3865–3882 (2018).

11. Islam, S. ul, Tang, Y. & Jackson, P. L. Asian monsoon simulations by Community Climate Models CAM4 and CCSM4. *Clim. Dyn.* **41**, 2617–2642 (2013).
